# Role models, compatibility, and knowledge lead to increased evolution acceptance

**DOI:** 10.1186/s12052-021-00155-x

**Published:** 2021-10-12

**Authors:** Daniel G. Ferguson, Jamie L. Jensen

**Affiliations:** Department of Biology, Brigham Young University, 4102 LSB, Provo, UT 84602 USA

**Keywords:** Evolution, Education, Role model, Religion, Reconciliation

## Abstract

**Background:**

Evolution acceptance is still low in the United States, especially among religious students. Due to low acceptance, researchers have used a wide variety of methods to increase evolution acceptance. Six culturally competent methods for teaching evolution to religious students have been identified, this manuscript looks specifically at the method of reconciliation between religion and evolution. The reconciliation module has been shown to effectively increase evolution acceptance while allowing students to maintain their religious views. However, we lack an understanding of why this method is effective. We measure evolution acceptance and religiosity at eight religiously affiliated institutions in the United States to again measure the effects of a reconciliation model in biology classrooms. This manuscript also attempts to address classroom influences that allowed students to reconcile evolution with their religious beliefs.

**Results:**

Of the eight schools that participated, there were no statistically significant decreases in the religiosity of the students over the semester. Five of the eight institutions had statistically significant increases in their evolution acceptance scores over the semester. We identified three major influences students mentioned as reasons for change towards evolution acceptance: the presence of a role model, discussions on religion and science compatibility, and learning about evolution.

**Conclusions:**

We identified influential practices instructors could integrate into their classrooms to help students better incorporate evolution into their personal views. Having a role model and talking about compatibility between religion and evolution are influential in changing students’ views about evolution. Learning the mechanisms of the theory of evolution is also important in changing students’ views about evolution and might be more impactful when used in conjunction with a role model or a compatibility discussion.

**Supplementary Information:**

The online version contains supplementary material available at 10.1186/s12052-021-00155-x.

## Background

Although evolution is considered the central idea in biology (Dobzhansky 1973; Brownell et al. 2014; American Association For The Advancement of Science 2011), 40% of adults reject that humans evolved with or without the presence of a Deity (Miller 2006; Gallup 2019). Low evolution acceptance also affects the general public and many high school and college students throughout the United States. Studies suggest that students’ religiosity influences their evolution acceptance (Barnes et al. 2017a; Manwaring et al. 2018), and many religious students feel evolution contradicts their beliefs (Cobern 1994; Dagher and BouJaoude 1997; Schilders et al. 2009; Coyne 2012). Some scientists think science can only progress if students leave their religious views behind(Coyne 2012,2016; Harris 2005; Dawkins and Ward 2006; Krauss 2015), whereas other scientists think a reconciliation between science and religious/world views among students is a more progressive approach to move science forward (Cobern 1994; Cobern 1996; Southerland and Scharmann 2013; Glaze et al. 2015; Barnes et al. 2017b; Lindsay et al. 2019; Tolman et al. 2020). In some cases, instructors educate students to align with the instructor’s views while ignoring the students’ views. While many other instructors help students see alternative viewpoints and allow students to choose for themselves. With the majority of the United States (75%) and college students (~ 66%) identifying as religious (Barnes et al. 2017b; Pew. 2015a,b), how educators choose to teach their classes regarding topics, like evolution, that are viewed by some as controversial may influence how religious students interact with these topics.

### Pedagogical methods influence evolution acceptance

There are many different approaches to teaching evolution with the goal of increasing student acceptance. One such method that appears to be ineffective is to approach the students assuming that their lack of acceptance is due to a deficit in knowledge of evolution or a deep misunderstanding of the facts (Lawson and Weser 1990; Honey 2015). In this “deficit approach” the teacher might discuss several non-scientific alternatives to evolution and show how science provides evidence of their error (Farber 2003). In this case, they are addressing religion, (Alexander et al. 2002), though with the intention to debunk and reject those ideas that lack scientific substance or evidence (Lilienfeld 2005). Consequently, rejecting religious or cultural beliefs as evidence of a deficit may lead to a personal conflict, which may diminish learning and decrease evolution acceptance among students (Cobern 1994; Dagher and BouJaoude 1997; Downie and Barron 2000; Stanger-Hall and Wenner 2014). Thus, while teaching the evidence can be beneficial, assuming a deficit in students is not an effective way to increase acceptance and anecdotally seems unproductive in changing opinions on evolution acceptance (Lindsay et al. 2019), especially among those who have rejected evolution due to perceived religious conflict (Nelson 1986).

A different approach to increasing evolution acceptance focuses on correcting common misconceptions of evolution and teaching correct evolutionary principles, leading to better understanding and therefore acceptance of evolution without assuming a deficit (Cherif et al. 2001; Farber 2003; Yerky and Wilczynski 2014). The results of teaching correct evolutionary principles are promising. Although some researchers have found no correlation between knowledge and acceptance (Bishop and Anderson 1990; Sinatra et al. 2003; Brem et al. 2003; Mead et al. 2017), more recent and sophisticated studies have found a positive correlation (Glaze et al. 2015; Rutledge and Warden 2000; Dunk 2019; Rissler et al. 2014; Weisberg et al. 2018). However, due to the complexity of evolution acceptance (Rutledge and Warden 2000; Wiles and Alters 2011; Winslow et al. 2011) it seems that only focusing on the facts or claiming deficit as a means to increase evolution acceptance sweeps the actual problem under the rug; students want their beliefs heard and validated (Scharmann 2005; Bertka et al.^2019^).

Research suggests other, more culturally-competent ways of teaching evolution that address the religious conflict may be more effective at increasing evolution acceptance in religious students than those methods previously mentioned. This research is highlighted in Barnes et al. (2017b), in their Religious Cultural Competence in Evolution Education (ReCCEE) paper, where they highlighted six essential culturally competent techniques for teaching evolution to students, specifically to those that hold religious beliefs. Studies on teaching students the nature of science (NOS) have shown increases in evolution acceptance among students (Cofré et al. 2018; Scharmann 2018; Barnes et al. 2019; Nelson et al. 2019) as they focus on the bounded nature of what science can and cannot answer. Although some scientists claim science can disprove God (Dawkins and Ward 2006; Coyne 2016), most scientists agree that science does not address supernatural entities (Gould and Gould 2011). One study found that religious students who viewed science as agnostic were more accepting of evolution than religious students who viewed science as atheistic (Barnes et al. 2020a). Therefore advocating science as agnostic instead of atheistic in our science classrooms may better allow students to differentiate between learning in science and the ways of learning in religion, without diminishing their religious identity.

Teaching to allow students to maintain their religious beliefs or worldviews may be an effective way to decrease evolution conflict and increase evolution acceptance among religious students. For example, in a study by Holt et al. (2018), they found that the presence of a role model (one who accepts evolution and is religious) in a biology classroom decreased perceived conflict between evolution and students’ worldviews while increasing their knowledge of evolution. Another study found that allowing students to have student-centered discussions in class allowed them to work out their thoughts about evolution and find a means to reconcile it with their own beliefs (Scharmann 2015). Thus, having a role model present in a class or allowing for student-centered discussions may help students work through perceived conflicts with evolution.

Another potentially effective way of teaching evolution to students is to offer them a way to reconcile religious beliefs with evolutionary theory (Barnes et al. 2017a; Lindsay et al. 2019; Manwaring et al. 2015). A reconciliation module for teaching evolution focuses on helping students bridge gaps between their religious beliefs and evolution by offering potential compatibility between a students’ religious beliefs or worldviews and evolutionary theory. Reconciliation modules may include activities such as class discussions on the compatibility between religion and science or why students might feel discomfort when talking about evolution. These discussions have increased evolution acceptance among students in biology classes (Truong et al. 2018) while allowing students to remain strong in their religious beliefs (Lindsay et al. 2019; Manwaring et al. 2015) over a semester. The reconciliation approach has been successful in other classes as well. One study (Tolman et al. 2020) compared a reconciliation discussion about evolution in a theology class with a biology class. They found statistically significant increases in evolution acceptance among students in both classes. Although they found more statistically significant gains in the biology class, this shows that using more culturally competent methods, even in a theology class, effectively bridges the gap between religious beliefs and evolutionary theory. However, the underlying theory that explains the benefits of this kind of education is still undetermined. We currently lack an understanding of why these teaching methods effectively increase evolution acceptance among students.

### Theoretical rationale

Cultural Border Crossing, an idea recently mentioned in the evolution education literature, potentially explains the benefits of a culturally competent approach to teaching evolution. Cultural Border Crossing (CBC) comes from cultural anthropology, in which learning science means obtaining the culture of science (Maddock 1981; Wolcott 1991). Culture, in this case, is defined as a set of beliefs, values, expectations, and conventional actions of a group (Geertz 1973; Aikenhead 1996; Aikenhead and Jegede 1996). CBC works in conjunction with Collateral Learning (CL), the cognitive mechanism of learners constructing two sets of concepts alongside each other. CBC/CL is similar to the reconciliation model described above in that the end goal is helping students find compatibility between two seemingly different ideas such as religious belief and evolutionary theory. But the CBC/CL framework highlights how some students easily move between cultures, whereas other students struggle to move between cultures. One highlighted reason religious students may struggle moving between a scientific culture and a religious home culture, is the perceived conflict between God, their religious beliefs, and the theory of evolution (Barnes et al. 2017a; Winslow et al. 2011; Barnes et al. 2020b). And recently a perceived conflict between God and religious beliefs and the theory of evolution has been shown to be the strongest predictor of evolution acceptance when entering a classroom (Barnes et al. 2021). Although we do not precisely measure our intervention using the CBC/CL framework or by measuring perceived conflict, they may be interesting to discuss later on, especially among those who found a way to reconcile their religious beliefs with evolutionary theory.

In this study, we aimed to explore reasons why students found our culturally component methodology to be effective at increasing their acceptance of evolution in biology classrooms (See (Barnes et al. 2017b; Lindsay et al. 2019) for more details), in eight religiously affiliated institutions. Our first purpose was to determine whether a reconciliation module is functional among different religious populations. This reconciliation module has previously been shown to effectively increase evolution acceptance while maintaining religiosity (Lindsay et al. 2019; Tolman et al. 2020; Manwaring et al. 2015). We hypothesize that these eight student populations will follow a similar pattern as previous studies, and we predict an increase in evolution acceptance while students’ religiosity remains constant. Second, we sought to understand what influenced the change in students' views of evolution, i.e., why this reconciliation module is successful. We gathered additional survey data to this end. We hypothesize that there are key components of the reconciliation module that highly influence students’ evolution acceptance. We predict that students will elucidate these key components and that these findings will better inform our pedagogical practices.

## Methods

### Informed consent

We obtained permission for this study from each of the institution’s institutional review boards IRB2021-074. Students were informed of the research and gave their consent to participate.

### Sample population

To determine the effectiveness of a reconciliation module and determine causal mechanisms among religious students, we surveyed college students from eight religiously affiliated institutions: Brigham Young University-Hawaii (BYUH) and Brigham Young University-Provo (BYUP) (Private schools affiliated with the Church of Jesus Christ of Latter-day Saints), Belmont University (BU; a private interdenominational Christian University), Hampden Sydney College (H-SC; a private men’s college affiliated with the Presbyterian Church), King University (KU; a private school affiliated with the Evangelical Presbyterian Church), Morgan State University (MSU; a public historically black school with a high volume of methodists), Northwestern College (NWC; a private school affiliated with the Reformed Church in America), and Southern Virginia University (SVU; a private school not affiliated with any religion but embraces values of the Church of Jesus Christ of Latter-day Saints). We had a total of 327 students participate in this research.

We choose these universities due to the high religiousness of the student populations and the faculty’s willingness to gather data. In addition, the instructors taught classes from introductory biology courses for non-majors and majors. We collected data in the fall 2019 and spring 2020 semesters. The amount of survey participation varied (See Table 1) depending on how the instructor incentivized the survey.

**Table 1:** Percentage of Completed Surveys. The table shows the number of completed pre and post-surveys at each institution

Schools	Completed pre surveys	Total pre/post surveys	Participation rate
BU	27	19	70.3%
BYUH	59	51	86.4%
BYUP	64	52	81.3%
HSC	64	34	53.1%
KU	26	16	61.5%
MSU	40	19	47.5%
NWC	148	65	43.9%
SVU	94	71	76%
*Total*	552	327	

### Building reconciliation modules

The universities that participated were recruited from attendees of our second HHMI-funded “Roads to Reconciliation” workshop that brought together institutional teams consisting of university theologians or religious scholars, university biologists, and a local community pastor to determine the effects of the reconciliation modules on acceptance and religiosity throughout the United States. A summary of the workshop is found in Lindsay et al. (2019). We had 17 institutions participate in the workshop, and eight of those institutions chose to participate in this study. The workshop’s goal was to encourage discussion regarding religious views and evolutionary theory and give teams a chance to create a lesson plan using the reconciliation module to be taught and used at each institution. The details of what was in the module and the duration of the module were not prescribed, so differences in duration and material discussed are different between institutions. However, the common focus of each module was to offer compatible possibilities between religious beliefs and evolutionary theory. To ensure enough commonality for comparison of results, we provided teams with an outline of the minimum requirements for the module. These include cultural barriers to consider, establishing a respectful environment in the classroom, a pre-class assignment, a procedure for offering reconciliation, a post-class assignment or follow-up, and resources. This outline is available in the Additional file 1.

The average time instructors spent using a reconciliation module in their classes for this study was 146 min, according to their reconciliation modules. Most of the instructors taught their modules in under 100 min over two class periods. All institutions mentioned in their modules the use of group or class discussions pertaining to their religious beliefs and evolutionary theory, along with optional or assigned readings from within their own religious sects. Our website (Reconciling Evolution 2021) has the teaching modules from each religion and institution that attended the workshop for those interested in more details.

### Quasi-experimental design

To determine the effect of the reconciliation modules on evolution acceptance and religiosity, we collected quantitative data (surveys) before and after evolution was discussed in their classes. The surveys gauged the religiosity of the students and their acceptance of evolution. In addition to determining the effects of the reconciliation modules on students, we also asked students a series of survey questions to determine why the reconciliation modules changed their views. BYUP was the only school able to collect this additional survey data on mechanisms.

### The measure of religiosity

Before and after evolution instruction, we surveyed students’ religiosity. This instrument was validated in a previous study on undergraduates at the primary author’s institution; CFI = 0.970, TLI = 0.977; RMSEA = 0.018 (Manwaring et al. 2015). A confirmatory factor analysis (CFA) using our data shows acceptable fit to the proposed model, given correlation of residual errors between religious influence items 1 and 2 (“How much influence do your religious beliefs have on what you wear?” and “How much influence do your religious beliefs have on what you eat and drink?”), and 3 and 4 (“How much influence do your religious beliefs have on your choices about whom you associate with?” and “How much influence do your religious beliefs have on what social activities you undertake?”), CFI = 0.921, TLI = 0.902, RMSEA = 0.082, SRMR = 0.055. The instrument consists of 15 questions on a six-point Likert scale that assesses self-reported religious practice (e.g., how often you attend church), religious influence (e.g., religion’s influence on what you wear), and religious hope (e.g., your belief in the afterlife). We calculated the total religiosity by summing the responses of the 15 items for a total of 90 points.

### The measure of evolution acceptance

To measure students’ evolution acceptance changes over the semester, we used the Inventory of Student Evolution Acceptance (ISEA). This instrument was created and validated on a sample of high school and college students; cronbach’s alpha for micro, macro, and human subscales were 0.96, 0.92, and 0.93, respectively (Nadelson and Southerland 2012). We further validated it using CFA. To achieve acceptable model fit, we had to remove all reverse coded items and other redundant questions.. Fit statistics were acceptable, CFI = 0.937, TLI = 0.919, RMSEA = 0.077, SRMR = 0.071 We used the I-SEA because of the uniqueness of its design. The I-SEA does not just measure evolution acceptance; it measures the acceptance of microevolution (e.g., I think there is an abundance of observable evidence to support the theory describing variation within a species), macroevolution (e.g., I think that new species evolved from ancestral species), and human evolution (e.g., I think that humans evolve). The modified ISEA instrument consists of 12 total questions on a 5-point Likert scale for 60 possible points. Since this instrument can also be used as three separate instruments (Barnes et al. 2019), we also treated each (microevolution, macroevolution, and human evolution) as a different survey. Thus, each survey consists of four questions on a 5-point Likert scale, for a total of 20 points for each subscale.

### The measure of student influences

To understand why students perceive a reconciliation module to be effective, we asked students at BYUP to rate whether a particular activity from class or outside of class was influential in their change in evolution acceptance. We used content validity evidence based on prior cultural competence studies to find if particular cultural competence activities influenced students’ views about evolution over the semester (Barnes et al. 2017b; Lindsay et al. 2019; Holt et al. 2018; Scharmann 1990). Students were asked eight questions on a six-point Likert scale from “very strongly influenced” to “no influence” (see Fig. 3 for what questions we asked students). Students took this survey after all evolution instruction of the class was taught, and data was only collected at one institution during the spring 2019 and fall 2019 semester (BYUP; *n* = 423). Thus, the data may only give insight into the views of students from this population. Nonetheless, these data will be a good starting point for other institutions if they desire to run a similar study with their students.

### Statistical analyses

To determine the effects of the reconciliation modules on acceptance and religiosity, we used a Wilcoxon signed-rank test to compare students’ pre-religiosity scores and post-religiosity scores and their pre-evolution acceptance scores and post- evolution acceptance scores. We only report descriptive statistics for the student influence survey since this was only collected at a one-time point at one institution.

## Results

### Religiosity

**Fig. 1:**
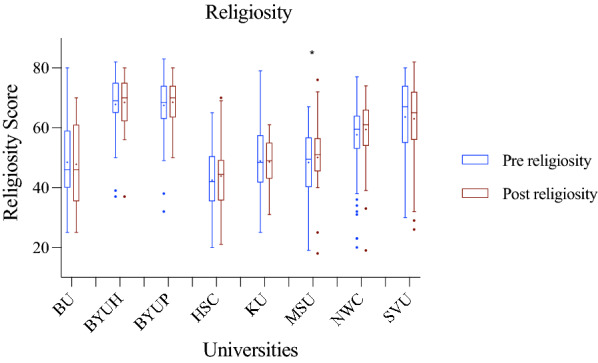
Student Religiosity Scores students. n = 327 students. Compares pre and post Religiosity scores between the religious institutions.

To determine whether our intervention affected students’ self-reported religiosity, we measured religiosity before and after evolution instruction. The religiosity instrument had a total score of 90 points. We used the non-parametric Wilcoxon signed-rank test to analyze this data. Seven of the eight participating institutions showed no statistically significant differences between pre and post religiosity scores at these schools (see Fig. 1; Table 2 for statistics). Whereas (MSU) had a statistically significant increase in their median religiosity score over the semester, meaning they became more religious over the semester.

**Table 2:** Statistical Results of Religiosity change. Shows the pre and post median religiosity scores, z-scores, and p-values of the schools

	Religiosity			
Schools	Pre	Post	t/z score (df)	p-value
BU	46	46	− 1.088	0.276
BYUH	68.7	68.3	.39 (50)	0.698
BYUP	68.9	68.5	.619 (50)	0.539
H-SC	42.6	44.3	− 1.094 (30)	0.283
KU	48.5	49	− 1.54	0.133
NWC	58.5	59.2	− 1.048 (60)	0.299
MSU	49.5	51	− 2.363	0.018
SVU	63.7	63	0.857 (70)	0.394

### Evolution acceptance

**Fig. 2:**
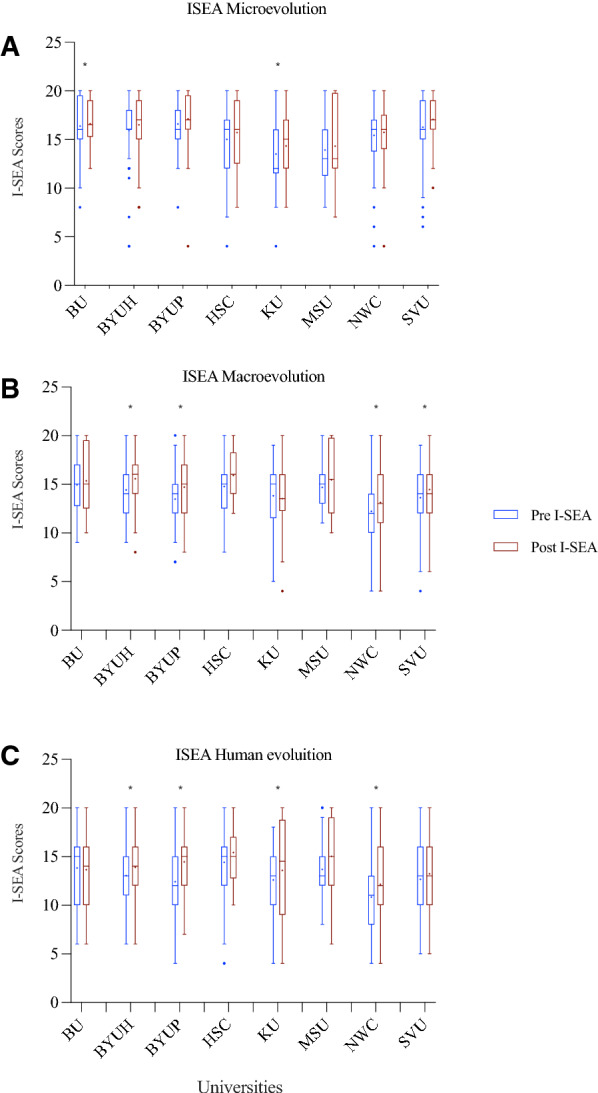
Student Evolution Acceptance Scores. n = 327 students. **A** compares pre and post microevolution scores. **B** Compares pre and post macroevolution scores. **C** compares pre and post human evolution scores

The I-SEA was used to measure students’ evolution acceptance before and after our intervention. We used the I-SEA as three separate surveys: microevolution, macroevolution, and human evolution. Each survey was four questions on a five-point Likert scale in which the sum of each instrument was 20 total points. Two out of the eight institutions (BU and KU) showed a statistically significant increase in microevolution acceptance scores over the semester (see Fig. 2; Table 3 for statistics). Four out of the eight institutions (BYUH, BYUP, NWC, SVU) showed a statistically significant increase in macroevolution acceptance scores over the semester. Four out of the eight institutions (BYUH, BYUP, KU, NWC) showed a statistically significant increase in human evolution acceptance scores over the semester. H-SC and MSU experienced no statistically significant changes in any of the instruments over the semester.

**Table 3:** Shows the pre and post median, z-scores, and p-values of the schools seperated into each I-SEA construct (microevolution, macroevolution, and human evolution)

Schools	Microevolution				Macroevolution				Human evolution			
	Pre	Post	*z score (df)*	*p*-value	Pre	Post	z score (df)	*p*-value	Pre	Post	z score (df)	*p*-value
BU	16	16	2.352 (19)	0.019	15	15	1.575 (19)	0.115	15	14	1.429 (18)	0.153
BYUH	16	17	1.202 (50)	0.229	14	16	3.087 (50)	0.002	13	14	2.535 (48)	0.011
BYUP	16	17	1.428 (50)	0.153	14	15	3.400 (51)	< 0.001	12	15	4.973 (52)	< .001
H-SC	16	16	1.714 (32)	0.087	15	16	1.709 (33)	0.088	15	15	1.486 (32)	0.137
KU	12	16	2.019(13)	0.043	15	13.5	0.722 (14)	0.47	13	14.5	2.129 (14)	0.033
NWC	16	16	1.307 (64)	0.191	12	13	2.767 (64)	0.006	11	12	2.852 (61)	0.004
MSU	13	13	− 1.188 (17)	0.235	15	15.5	− 0.528 (18)	0.597	13	15	1.352 (17)	0.176
SVU	16	17	0.617(70)	0.537	14	14	2.163 (69)	0.031	13	13	1.127 (69)	0.26

### Influences of change in evolution acceptance

**Fig. 3:**
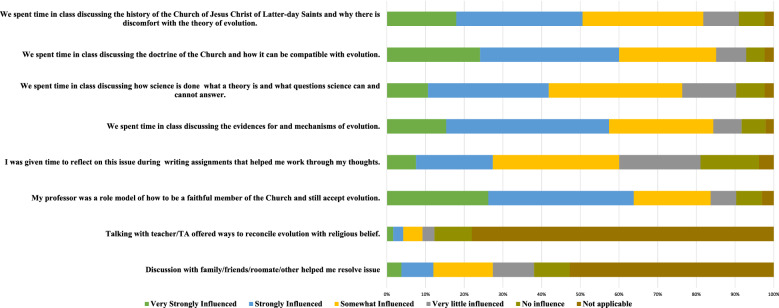
Student Influence for Change on Evolution Acceptance

We asked additional questions of students at BYUP to determine which aspects of the reconciliation module most influenced students’ opinions on evolution over the semester. These questions allow us to see what is most important for students struggling with evolution (see Fig. 3). Sixty-four percent of the students said they were very strongly influenced or strongly influenced by having a role model who was a faithful member of their church. At the same time, 60% of students were very strongly influenced and strongly influenced by the time spent discussing church doctrine and its compatibility with evolution. Fifty-seven percent of students were very strongly influenced and strongly influenced by discussing evolution’s evidence and mechanisms. Just over 50% of the students were very strongly influenced and strongly influenced by discussing their religions’ history, and why they may feel discomfort. At the same time, 42% found a discussion of the nature of science influential. Five percent of the students found talking to the teacher or TA outside of class very strongly and strongly influenced their views on evolution. Twelve percent of students said discussing evolution with their family or friends was also influential in changing their views about evolution.

## Discussion

Our results aligned with our first hypothesis that using a reconciliation module in an introductory biology class shows gains in evolution acceptance at most institutions among religious students without diminishing or negatively affecting students’ religiosity. We also were able to determine critical factors that reveal why a reconciliation module is so important for religious students learning about evolution. This was done by surveying students about potential practices that influenced their evolution acceptance over a semester.

### Minds can change, but religiosity remains

Our first hypothesis is supported in that a reconciliation module shows increases in evolution acceptance without decreases in religiosity, which aligned with our prediction. Religious students who enter science classes with the idea that evolution is not compatible with Judeo-Christian beliefs can change their minds when the perceived conflict between creation and evolution is presented in a reconciliatory way. Our participants took the I-SEA survey (Nadelson and Southerland 2012), which measures acceptance of microevolution, macroevolution, and human evolution. No schools showed statistically significant increases in all areas of the I-SEA, whereas some schools had statistically significant increases in two areas of the I-SEA—BYUH, BYUP, and NWC (macroevolution and human evolution) and KU (microevolution and human evolution). H-SC and MSU had no statistically significant changes in any areas of their I-SEA scores (see Fig. 2). Lack of complete survey data could account for the non-significance in our results. Interestingly, schools that did not have significant increases in evolution acceptance still showed positive increases in all areas of the I-SEA (see Table 3). Considering that many Judeo-Christan believers tend to have conflicts with macroevolution and human evolution, these results are promising given that students were able to increase evolution acceptance and maintain their religious beliefs. It should be noted that instructors had a great deal of flexibility in the way in which their lessons were designed. Some of these differences may account for differences in overall effectiveness and are a limitation of our design. However, it should be encouraging that regardless of differences in curricula, and overall approach to offering students ways of reconciling evolution with religion is a successful approach to increasing evolution acceptance.

We observed no statistically significant changes in religiosity in seven of the eight institutions (see Fig. 1). The one institution that changed was MSU, which saw a statistically significant increase in religiosity over the semester. Interestingly, MSU was one of the three schools that did not significantly change evolution acceptance. Although most schools saw students’ acceptance of evolution scores increased, their religiosity scores remained the same. This confirms that students can keep their religious identity and still increase their acceptance of evolution. Students holding to their religious beliefs goes against what some scientists have claimed to be the main problem with evolution acceptance in the United States (Coyne 2012,2016). Our results, along with others (Barnes et al. 2017b, 2020a; Lindsay et al. 2019) disagree. Barnes and Brownell (2017b) call upon educators and scientists to practice culturally competent teaching methods to help bridge the gap between religious beliefs and secular views. These culturally competent teaching practices have decreased conflict between religion and evolution while increasing evolution acceptance. Using a reconciliation module in teaching evolution allows students to construct their own views about evolution while allowing them to reconcile this view with their religious beliefs.

### Influencers of evolution acceptance

We set out to investigate some leading factors that influenced students’ views about evolution. Our results showed the three most influential themes as indicated by students that changed their views about evolution are (1) having a role model present in class, (2) learning the mechanisms of evolution, and (3) talking about the compatibility of religious beliefs with evolution. In addition, many students were also very strongly influenced or strongly influenced by discussing the discomfort found within their religion’s history dealing with evolution (52%) and discussing the nature of science (42%). Many of these influences in changing students’ views on evolution have been discussed in the scientific literature. Knowing their importance with religious students may help educators better modify their teaching to incorporate these influences in their classrooms.

Having a role model, especially one who is religious and one who also accepts evolution, seems to be important for allowing students to change their views on evolution. Students in our study saw their professor as a role model who was firm in their religious beliefs and accepted the science of evolution. Students were able to see that evolution and religion could be reconciled because they saw a role model who found compatibility (Winslow et al. 2011). In one study, once students saw someone who reconciled evolution and religion, the conflict they felt with evolution decreased (Holt et al. 2018). Our results align with other studies as well (Barnes et al. 2017a; Winslow et al. 2011; Holt et al. 2018), which specify the positive impact a role model has on Judeo-Christian students and evolution acceptance. Whether the instructor acts as a role model or invites a guest lecturer to act as a role model, this appears to be able to significantly impact students’ ability to reconcile their beliefs with evolution.

Along with having a role model present, we also found that students felt that discussing the compatibility between religion and evolution was influential in helping them work out their views on evolution, which aligns with what others have seen with discussing compatibility in the classroom (Barnes et al. 2017a; Lindsay et al. 2019; Tolman et al. 2020; Wiles and Alters 2011; Scharmann and Butler 2015), and compatibility discussions even as short as six minutes are influential in changing students’ views about evolution (Truong et al. 2018). Thus, it may be important for religious students to find compatibility between religion and evolution. Students finding compatibility, whether it be through a role model or discussions about compatibility, may lead to a higher desire to learn about the evidence for and mechanisms of evolution, which almost 60% of our students reported as strongly influential in changing their views about evolution.

Learning about the mechanisms and evidence for evolution appears to be important for students’ understanding of the theory of evolution and for changing students’ minds about evolution. One study by Talbot et al. (Talbot et al. 2020), looked at biology majors taking their capstone evolution course and found that increased knowledge of the mechanisms and evidence for evolution was the most important factor for students’ increasing their acceptance of evolution while completing their coursework. In other studies, there was no correlation between teaching the facts of evolution and acceptance of evolution (Bishop and Anderson 1990; Sinatra et al. 2003; Brem et al. 2003; Mead et al. 2017). It has been suggested that evolution acceptance is a multi-faceted and complex system with many factors (Barnes et al. 2017b; Rutledge and Warden 2000; Wiles and Alters 2011; Winslow et al. 2011). It is possible that for facts and knowledge to be positively correlated with evolution acceptance, compatibility must first be attained by students. Once they found compatibility with evolution, students may have opened their minds to learning evolution, leading to an increased desire to learn. Since we did not measure knowledge of evolution, we cannot say for sure if this was the case in our sample. The change of compatibility with beliefs and evolution on the effects of evolution knowledge or even misconceptions may be interesting studies moving forward.

Teaching the nature of science has also been shown to be a potentially effective method in increasing evolution acceptance (Rutledge and Warden 2000; Scharmann 2018; Nelson et al. 2019; Carter and Wiles 2014; Dunk et al. 2017). The students in our study also reported it to be influential. Another influential factor that students reported was talking about discomfort found within students’ religious beliefs. In this case, students learned about the reasons for discomfort in their religious views by viewing authoritative statements, both for and against evolution, made within their religion’s history. Letting students view these statements and ponder them may have been useful in helping students overcome discomfort about evolution.

## Conclusion

Although we did not have statistically significant increases in evolution acceptance at all the schools, we did universally see at least small increases towards acceptance. We also identified potentially influential practices instructors could integrate into their classrooms to help students better incorporate evolution into their personal views, which hopefully leads to better evolution acceptance. Having a role model and talking about compatibility between religion and evolution, and learning the mechanisms of the theory of evolution may be important in changing students’ views about evolution. If instructors feel uncomfortable acting as role models or having a discussion about religious compatibility and evolution, they can seek help from their community to find someone (e.g., a religious pastor, a community leader) who may be willing to assist them as a guest lecturer. Our research suggests that allowing students to find compatibility between religious beliefs and evolutionary theory is important for religious students in decreasing their conflict while increasing their acceptance and knowledge of evolution.

## Supplementary Information

**Additional file 1**. Participants used the outline to create a reconciliation module at their respective institutions.

## Data Availability

Data will be freely available.

## Abbreviations

ReCCEE: Religious Cultural Competence in Evolution Education
BU: Belmont University
BYUH: Brigham Young University-Hawaii
BYUP: Brigham Young University-Provo
CBC: Cultural Border Crossing
CL: Collateral Learning
H-SC: Hampden Sydney College
KU: King University
MSU: Morgan State University
NWC: Northwestern College
SVU: Southern Virginia University
