# Supplementary material for: Role models, compatibility, and knowledge lead to increased evolution acceptance
Source: Evolution (N Y). 2021 Oct 12;14(1):16. doi: 10.1186/s12052-021-00155-x (PMC8550684; doi:10.1186/s12052-021-00155-x)
Supplement: Supplementary file 1 — Additional file 1. Participants used the outline to create a reconciliation module at their respective institutions. [file 12052_2021_155_MOESM1_ESM.docx]

**Reconciling Evolution & Religion**

**A (enter your faith tradition here) Perspective**

Instructor Guide

**BACKGROUND INFORMATION**

**Learning Outcomes**

What do you want students to be able to do when they are done?

**Type of Course**: Title, lower or upper division, majors or non-majors

**Suggested Location in the Curriculum**: Where in the curriculum should this lesson be taught?

**Estimated Time**: How long does this lesson take?

**Advanced Preparation for Instructor**: What should the instructor do, read, watch before teaching this lesson to prepare them?

**Supplies Needed**: What supplies are needed to run this activity?

**Cultural Barriers to be Considered**: What are the cultural barriers to evolution that are most prevalent in your faith tradition? Where do they come from (doctrinally? culturally? socially?)? What specific issues with evolution are students likely to have?

**Establishing Respect in the Classroom**: How will you establish a healthy classroom environment to have this conversation?

**OUTLINE OF RECONCILIATION ACTIVITIES**:

**Pre-Class Assignment(s)**: Is there anything you would like students to do to prepare them for this conversation?

**Procedure**

- This portion should contain a step-by-step guide to instructors on how to run the activity

Consider including sub-headings that describe your approach (e.g., *Acknowledging the existence of conflict, Addressing the difference between science and religion, Addressing creation from a religious standpoint,* etc.)

**Post-Class Assignment**: Is there anything you would like students to do after class to further process this information?

**AVAILABLE RESOURCES FOR STUDENTS AND INSTRUCTORS**

What resources are available for both students and instructors to consult to learn more about the intersection of evolution and your faith tradition?
